# Hypochlorous Acid-Generating Electrochemical Catheter Prototype for Prevention of Intraluminal Infection

**DOI:** 10.1128/Spectrum.00557-21

**Published:** 2021-10-27

**Authors:** Edison J. Cano, Laure Flurin, Abdelrhman Mohamed, Kerryl E. Greenwood-Quaintance, Yash S. Raval, Haluk Beyenal, Robin Patel

**Affiliations:** a Division of Infectious Diseases, Mayo Clinic, Rochester, Minnesota, USA; b Division of Clinical Microbiology, Mayo Clinic, Rochester, Minnesota, USA; c The Gene and Linda Voiland School of Chemical Engineering and Bioengineering, Washington State University, Pullman, Washington, USA; Memorial Sloan Kettering Cancer Center

**Keywords:** catheter-related bloodstream infection, hypochlorous acid, electrochemistry, infection prevention

## Abstract

Central line-associated bloodstream infection (CLABSI) contributes to mortality and cost. While aseptic dressings and antibiotic-impregnated catheters prevent some extraluminal infections, intraluminal infections remain a source of CLABSIs. In this proof-of-concept study, an electrochemical intravascular catheter (e-catheter) prototype capable of electrochemically generating hypochlorous acid intraluminally using platinum electrodes polarized at a constant potential of 1.5 electrode potential relative to saturated silver/silver chloride reference electrode measured in volts (V_Ag/AgCl_) was developed. After 24 h of prepolarization at 1.5 V_Ag/AgCl_, their activity was tested against clinical isolates of Staphylococcus aureus, Staphylococcus epidermidis, Enterococcus faecium, and Escherichia coli derived from catheter-related infections. e-catheters generated a mean HOCl concentration of 15.86 ± 4.03 μM and had a mean pH of 6.14 ± 0.79. E-catheters prevented infections of all four species, with an average reduction of 8.41 ± 0.61 log_10_ CFU/ml at 48 h compared to controls. Polarized e-catheters which generate low amounts of HOCl continuously should be further developed to prevent intraluminal infection.

**IMPORTANCE** Catheter-related infections constitute an economic and mortality burden in health care. Several options are available to reduce the risk of infection, but only a few focus on preventing intraluminal infection, which occurs in long-term catheters, most often used for dialysis, prolonged treatment, or chemotherapy. A prototype of a catheter called an “e-catheter” composed of three electrodes, capable of producing hypochlorous acid (HOCl) electrochemically in its lumen, was developed. When polarized at 1.5 V, chloride ions in the solution are oxidized to continuously produce low amounts of HOCl, which exhibits antibacterial activity in the lumen of the catheter. Here, this prototype was shown to be able to generate HOCl as well as prevent infection in a preliminary *in vitro* catheter model. This approach is a potential strategy for catheter infection prevention.

## INTRODUCTION

Intravascular catheters are essential devices in health care for a wide range of applications. They are particularly needed in critically ill patients (e.g., for fluid administration or resuscitation) but have also been increasingly used in noncritically ill patients for long-term medication delivery, hemodialysis, or parenteral nutrition administration (1). Unfortunately, conventional catheters pose a direct route of entry for bacteria to the bloodstream, resulting in central line-associated bloodstream infection (CLABSI). CLABSI contributes to excess health care expenses in the United States, with up to US $90,000 per patient and excess mortality of 15% to 25% per episode (2). In 2019 alone, the U.S. Centers for Disease Control and Prevention reported more than 30,000 CLABSI events in the United States (3), although underreporting is a concern with these figures (4).

The pathophysiology of CLABSI comprises two main routes of infection, the extraluminal route for short-term central venous catheters (CVCs), where microorganisms enter from the insertion site and colonize the catheter tip, and the intraluminal route for long-term catheters (planned for >5 days duration), where frequent manipulation of the line results in introduction of pathogens into the lumen (5, 6). Many available technologies developed for CLABSI prevention focus on aseptic techniques to mitigate extraluminal infections. For instance, use of chlorhexidine-impregnated dressings (7) or externally impregnated chlorhexidine/silver sulfadiazine catheters has shown a decrease in CLABSI (8). However, intraluminal infections remain a major source of CLABSI, particularly for long-term catheters, with intraluminal bacterial colonization rates as high as 40% in lines older than 30 days (9). Intraluminal strategies to prevent CLABSI include intraluminal antibiotic/antimicrobial agent locks, antimicrobial-impregnated catheter lumens, and antiseptic barrier caps. Intraluminal antibiotic solutions have shown a reduction in CLABSI rates, although this approach is prone to selection of bacterial resistance, side effects from contents infused systemically, and catheter damage due to high concentrations of these solutions (10, 11). Antiseptic barrier caps constitute one of the most widely implemented strategies to decrease intraluminal contamination from bacteria entering the catheter hub, although they are only active at the hub or injection site (12). Other techniques, such as silver-impregnated central venous catheters, have shown variable rates of CLABSI reduction and bacterial colonization but have overall not proved superior to conventional catheters (13, 14). Silver-platinum-carbon-impregnated catheters were shown to be inferior to rifampin-minocycline-coated catheters (15), despite the *in vitro* antimicrobial activity of these metals (16, 17). Another technology that delivers silver particles into the lumen, silver iontophoretic catheters, showed no benefit in comparison to regular catheters in a clinical trial (18). Although implementation of care bundles and enhancement of extraluminal/skin asepsis have decreased CLABSI rates, development of new approaches to prevent intraluminal infections is needed to further reduce CLABSI rates (9).

The most common pathogens found in CLABSI in the United States are coagulase-negative staphylococci and Staphylococcus aureus, which were resistant to methicillin in more than 50% of cases between 2011 and 2014 (19), followed by *Enterococcus* sp., *Candida* sp., and Gram-negative bacteria such as Escherichia coli, Klebsiella pneumoniae, and Pseudomonas aeruginosa (20). Up to 20% of CLABSIs are caused by multidrug-resistant (MDR) organisms; such infections are difficult to treat, targeted with a limited number of effective antibiotics, and associated with high mortality in intensive care units (21). Hence, there is a need for nonantibiotic alternative approaches.

In previous work, we demonstrated that HOCl can be continuously generated at low concentrations on the surface of inert conductive electrodes polarized at a constant potential (22). Briefly, HOCl can be generated on polarized electrodes through oxidation of chloride ions in solution. At electrode potentials (*E*°) above 1.138 electrode potential relative to saturated silver/silver chloride reference electrode measured in volts (V_Ag/AgCl_), chloride ions are oxidized to generate Cl_2_ gas (equation 1) (23). Dissolved chlorine rapidly hydrolyzes in water to form a mixture of HOCl and hypochlorite (equations 2 and 3).

(equation 1) $${\text{2Cl}}^{-}\to {\text{Cl}}_{\text{2}}+2e^{-}E{}^{\circ}=\text{ 1}.{\text{138 V}}_{\text{Ag}/\text{AgCl}}$$

(equation 2) $${\text{Cl}}_{\text{2}}+{\text{H}}_{2}\text{O}⇋{\text{Cl}}^{-}+\text{HOCl}+{\text{H}}^{+}$$

(equation 3) $$\text{HOCl}⇋{\text{H}}^{+}+{\text{OCl}}^{-}$$

Electrochemical HOCl generation could be applied as a strategy to prevent intraluminal catheter infections. However, such technology requires controlled generation of HOCl. In our previous work, we have demonstrated its efficacy in treating monomicrobial and polymicrobial biofilms formed by antibiotic-resistant organisms using an *in vitro* biofilm model (24). It was hypothesized that this concept could be applied in an *in vitro* catheter model in which HOCl is electrochemically generated at low continuous levels, such that it could prevent bacterial growth in the catheter lumen, thereby preventing infection. An electrochemical catheter (e-catheter) prototype consisting of a Tygon S3 E-3603 tube with three electrodes embedded within its lumen, two platinum wires as the working and counter electrodes, and a silver/silver chloride (Ag/AgCl) wire as a reference electrode was designed and evaluated. Once one of the platinum electrodes is polarized, HOCl is produced along the e-catheter’s intraluminal volume, from the hub to the tip.

In this study, the three objectives were (i) to develop a prototype *in vitro* e-catheter model, (ii) to verify electrochemical generation of HOCl in the prototype catheter’s lumen, and (iii) to preliminarily test its activity in an *in vitro* e-catheter prevention model with four bacterial isolates derived from catheter-related infections.

## RESULTS

### Electrochemical characterization.

**FIG 1:**
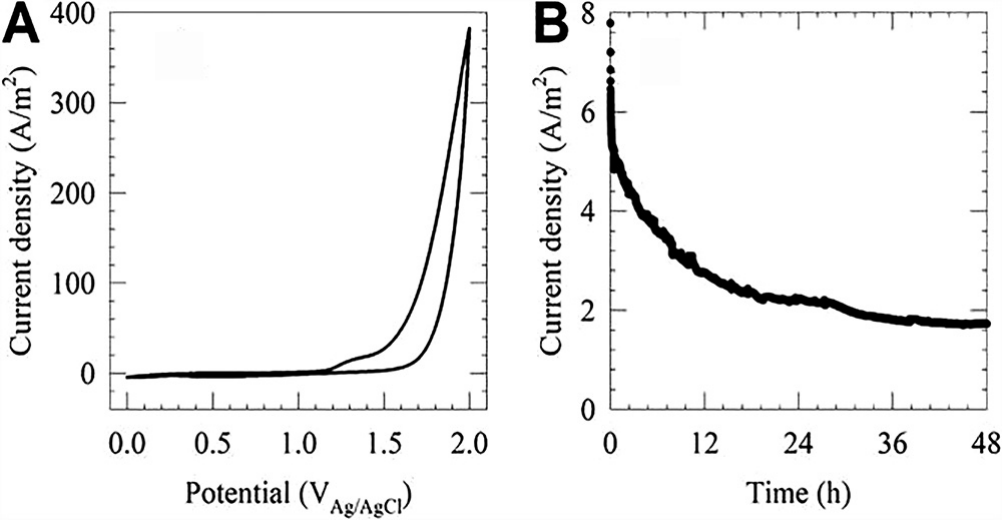
Representative data set showing a cyclic voltammogram of the e-catheter working electrode at a scan rate of 0.010 V/s (A) and a chronoamperometric scan with the working electrode polarized at 1.5 V_Ag/AgCl_ over 48 h (B).

Preliminary electrochemical experiments were performed to verify the region where anodic reactions occur in the e-catheter. In these preliminary experiments, the e-catheter was operated without inoculated bacteria. Cyclic voltammetry experiments showed background current levels when the electrode potential was between 0.0 V_Ag/AgCl_ and 1.1 V_Ag/AgCl_ (Fig. 1A). Anodic current was observed above the onset potential of ∼1.1 V_Ag/AgCl_. Anodic current increased with increasing potential, plateauing above ∼1.26 V_Ag/AgCl_ and then continuing to increase above ∼1.35 V_Ag/AgCl_. During treatment, e-catheters were polarized at a constant potential of 1.5 V_Ag/AgCl_. Figure 1B shows a representative chronoamperometric scan recorded while the working electrode was controlled at 1.5 V_Ag/AgCl_. The working electrode current started initially at 9.87 A/m^2^ and continued to decrease afterward, reaching 2.25 A/m^2^ after 5 h of polarization. The current density began to stabilize at 1.48 ± 0.08 A/m^2^ after 10 h of polarization, reaching 1.93 ± 0.19 A/m^2^ after 20 h.

### pH and HOCl measurements.

**FIG 2:**
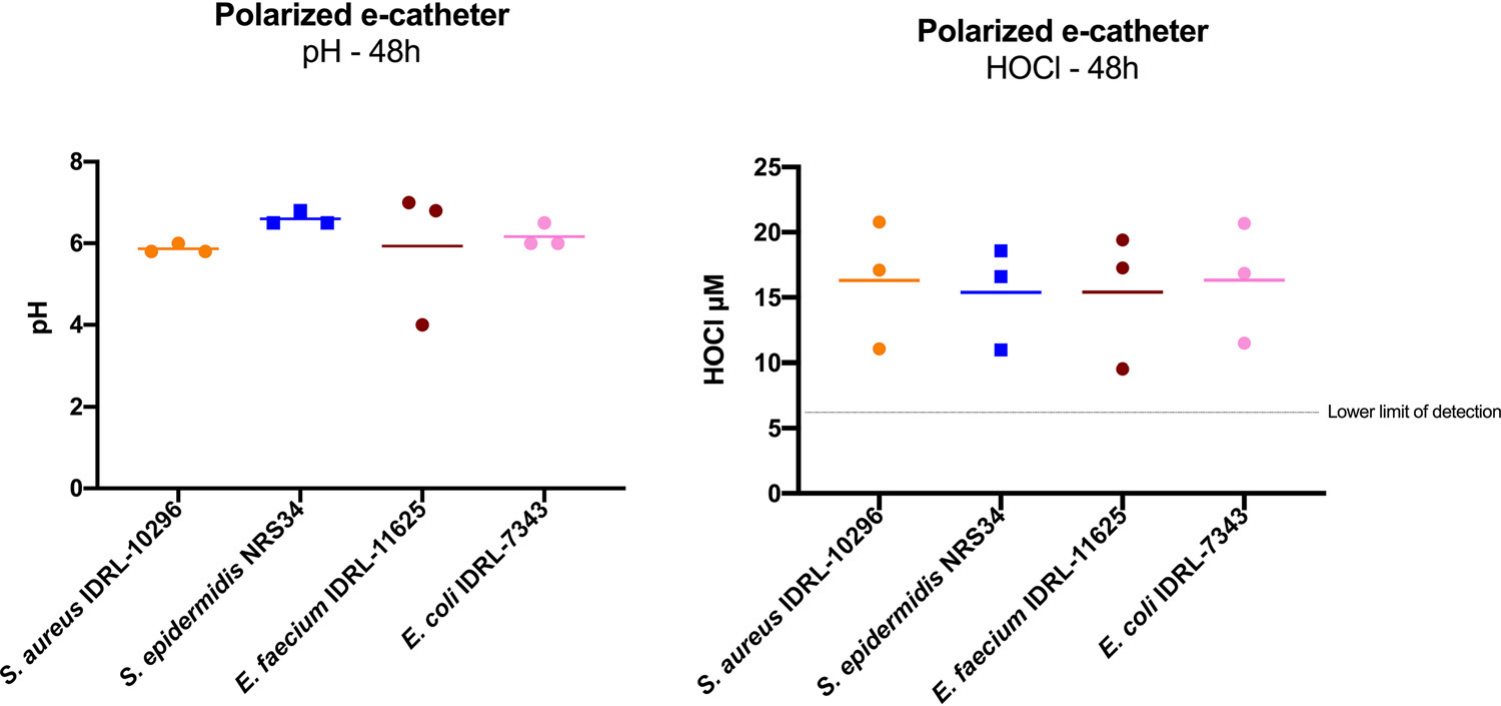
Measurement of pH and HOCl at 48 h in polarized e-catheters. Each dot represents a replicate; bars represent means.

The mean pH measured at 48 h in polarized e-catheters was 6.14 ± 0.79 (Fig. 2). One replicate of Enterococcus faecium IDRL-11625 had a short circuit in the e-catheter that led to a pH of 4. The HOCl concentration at 48 h was calculated based on free chlorine measurements. The mean concentration of HOCl across all isolates at 48 h in e-catheters was 15.86 ± 4.03 μM. The mean HOCl concentrations in polarized e-catheters infected with S. aureus IDRL-10296, Staphylococcus epidermidis NRS34, E. faecium IDRL-11625, and E. coli IDRL-7343 at 48 h were 16.31 ± 4.91 μM, 15.38 ± 3.94 μM, 15.40 ± 5.19 μM, and 16.33 ± 4.61 μM, respectively. Since the current was higher in the first two than at 48 h, HOCl concentrations were measured at 0.5, 1, and 2 h in a noninfected polarized e-catheter in triplicate. Mean HOCl concentrations were 123.45 ± 12.09, 152.07 ± 34.98, and 179.96 ± 5.64 μM at 0.5, 1, and 2 h, respectively. Detailed results are shown in Fig. S1 in the supplemental material.

### Preliminary assessment of e-catheter antimicrobial activity. (i) S. aureus IDRL-10296.

**FIG 3:**
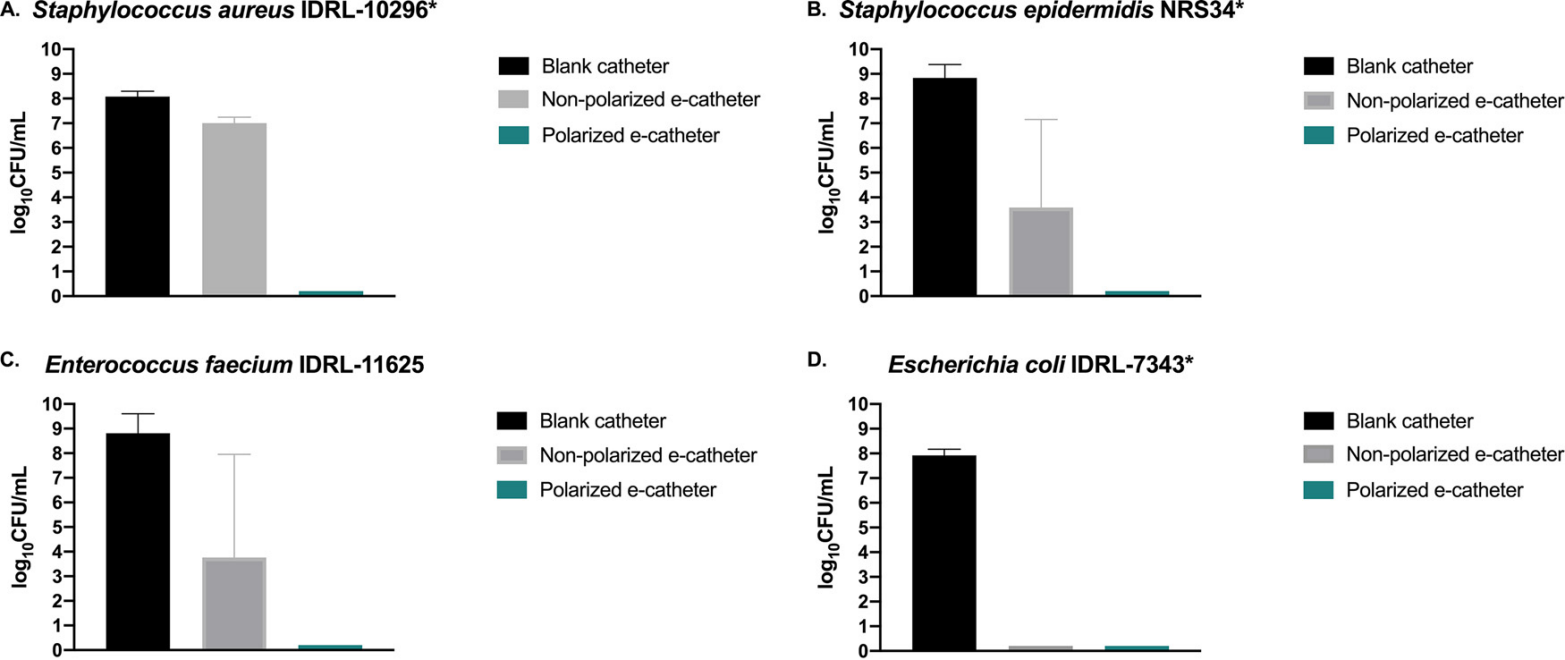
Prevention of infection after 48 h of polarization (24 h of infections) using e-catheters (polarized and nonpolarized) compared to blank catheters. Asterisks indicates statistically significant reductions in cell counts in polarized e-catheter compared to blank catheter groups (*P* < 0.05).

For S. aureus IDRL-10296, mean bacterial cell concentrations were 8.08 ± 0.22 log_10_ CFU/ml in blank catheters, 7.01 ± 0.24 log_10_ CFU/ml in nonpolarized e-catheters, and 0 ± 0 log_10_ CFU/ml in polarized e-catheters (*P* = 0.004). In comparison to blank catheters, the average reduction in bacterial cell concentration at 48 h was 1.07 ± 0.36 log_10_ CFU/ml for nonpolarized e-catheters and 8.08 ± 0.22 log_10_ CFU/ml for polarized e-catheters (Fig. 3A).

### (ii) S. epidermidis NRS34.

For S. epidermidis NRS34, after 48 h, mean bacterial cell concentrations were 8.84 ± 0.55 log_10_ CFU/ml in blank catheters, 3.57 ± 3.57 log_10_ CFU/ml in nonpolarized e-catheters, and 0 ± 0 log_10_ CFU/ml in polarized e-catheters (*P* = 0.014) (Fig. 3B).

### (iii) E. faecium IDRL-11625.

For E. faecium IDRL-11625, after 48 h, mean bacterial cell concentrations were 8.82 ± 0.79 log_10_ CFU/ml in blank catheters, 3.75 ± 4.19 log_10_ CFU/ml in nonpolarized e-catheters, and 0 ± 0 log_10_ CFU/ml in polarized e-catheters (*P* = 0.05) (Fig. 3C).

### (iv) E. coli IDRL-7343.

For E. coli IDRL-7343, after 48 h, mean bacterial cell concentrations were 7.92 ± 0.25 log_10_ CFU/ml in blank catheters and 0 ± 0 log_10_ CFU/ml in nonpolarized e-catheters and polarized e-catheters (*P* = 0.036) (Fig. 3D). Given that in nonpolarized and polarized e-catheters, bacterial cell concentrations were reduced to the limit of detection for E. coli IDRL-7343, the antibacterial effects of platinum electrodes alone and Ag/AgCl electrodes alone were compared in the catheter model using E. coli IDRL-7343 alongside S. epidermidis NRS34; it was found that the Ag/AgCl electrodes had an antibacterial effect. The Ag/AgCl effect on bacterial cell reduction was less for S. epidermidis NRS34 (3.49 ± 1.40 log_10_ CFU/ml) than for E. coli IDRL-7343 (8.74 log_10_ CFU/ml) (Fig. S2).

Polarized e-catheters reduced bacterial concentrations below the limit of detection for all isolates, with a mean reduction of 8.41 ± 0.61 log_10_ CFU/ml compared to blank catheters. Nonpolarized e-catheters had a variable effect, from minimal with S. aureus IDRL-10296 to maximal with E. coli IDRL-7343.

## DISCUSSION

Here, a novel intravascular catheter prototype, referred as an e-catheter, which generates HOCl intraluminally when polarized at a constant potential of 1.5 V_Ag/AgCl_, is described. Polarized e-catheters exerted antimicrobial activity, reducing viable cell counts below the limit of detection for S. aureus IDRL-10296, S. epidermidis NRS34, E. faecium IDRL-11625, and E. coli IDRL-7343 in an *in vitro* infection prevention model. HOCl concentrations were measured, confirming electrochemical HOCl generation via chloride oxidation on the surface of the polarized electrodes. The average HOCl concentration after 48 h of polarization was 15.86 ± 4.03 μM at a mean pH of 6.14 ± 0.79. In this range of pHs (5 to 6.5), HOCl is the predominant form, accounting for >90% of free chlorine in solution, with the remainder consisting of OCl^−^ ions (25). Ono et al. (26) showed that the bactericidal effect of HOCl was highest under weakly acidic conditions (pH 5.0 to 6.0), suggesting that e-catheters operate near optimum pH for antimicrobial effect.

Hypochlorous acid is generated from anodic oxidation of chloride ions (equations 1, 2, and 3). Electrons delivered through the working electrode oxidize chloride ions to chlorine (equation 1), which in turn dissociates to HOCl (equation 2) and hypochlorite (equation 3). The oxidation rate is measured as positive current, which is shown for 48 h in Fig. 1B. The current density is highest initially (between 7.8 and 4.5 A/m^2^ within the first 2 h) due to the capacitive response associated with changing the potential of the working electrode, in addition to chloride oxidation. The current density then continues to decrease (between 4.5 and 2.2 A/m^2^ between 2 h and 20 h), likely due to consumption of chloride ions near the surface of the electrode. Finally, current density stabilizes at 1.93 ± 0.19 A/m^2^ after 20 h due to equilibrium between the mass transport rates of chloride ions at the surface of the electrodes and the rates of chloride oxidation. Based on current measurements which are correlated with the HOCl generation rates, the e-catheter is active for at least 48 h, while the HOCl generation rate is highest at the start of polarization.

In previous work, Raval et al. (27) determined the MICs of HOCl for 27 isolates, including S. aureus, S. epidermidis, E. faecium, and E. coli. The MICs for 6 S. aureus and 3 S. epidermidis isolates ranged from 990 to 1,690 μM, and the MICs for E. faecium and E. coli isolates were 990 μM HOCl; concentrations above 286 μM are cytotoxic to mammalian cells (28). The described e-catheter prototype produced 179.96 ± 7.45 μM HOCl after 2 h of polarization. Continuous production of HOCl in the e-catheter prevented bacterial infection, even though the final concentration measured after 48 h of polarization was below the MIC. With MIC measurements, initial HOCl concentrations may decrease with time as HOCl reacts with planktonic bacteria. The described e-catheter overcomes such limitations by generating HOCl continuously at lower amounts corresponding to lower concentrations. Such continuous generation of low concentrations may apply consistent antibacterial pressure on potential pathogens.

For the four isolates tested, the average reduction in viable cell concentration was 8.41 ± 0.61 log_10_ CFU/ml, which is both statistically and potentially clinically significant. Although the antimicrobial effect of polarized e-catheters is postulated to be due to electrochemical generation of HOCl, alternative antimicrobial mechanisms, such as the previously described electricidal effect (seen with the application of direct electric current) (29), other active by-products of polarization, or the presence of an Ag/AgCl electrode, have yet to be systematically ruled out. Indeed, nonpolarized e-catheters showed antimicrobial activity against some bacterial isolates studied, even without electrical current application. The reduction in bacterial cell concentrations and the high standard deviation in the nonpolarized e-catheters infected with S. epidermidis NRS34 and E. faecium IDRL-11625 in comparison to blank catheters suggests an inconsistent antibacterial activity of nonpolarized e-catheters. On the other hand, with polarized e-catheters, the reduction in bacterial cells compared to blank catheters for those two isolates was consistent and clinically significant: 8.84 ± 0.55 log_10_ CFU/ml for S. epidermidis NRS34 and 8.82 ± 0.79 log_10_CFU/ml for E. faecium IDRL-11625.

Antimicrobial activity was documented in the presence of the Ag/AgCl reference but not platinum electrodes (see Fig. S2 in the supplemental material). Ag/AgCl has shown antimicrobial activity against Gram-negative bacteria (e.g., E. coli, K. pneumoniae, P. aeruginosa), achieving significant bacterial cell reductions in less than 8 h (30). Gram-positive bacteria (S. aureus and Streptococcus equi) require longer exposure times to achieve significant bacterial cell reductions, consistent with findings with nonpolarized e-catheters infected with S. aureus IDRL-10296. This presents a limitation to the model; future work will explore a replacement or modification of the Ag/AgCl electrode.

There are several limitations to the described work. Intraluminal fluid was studied; it is possible that the bacteria formed biofilms on the walls of infected e-catheters and/or the wires. Methods which sample biofilms should be used in future studies. Another limitation of this work is the design of the e-catheter. In the described prototype, the wires are in the lumen. Future development may focus on applying wires directly to catheter walls to minimize impedance to fluid flow. After further development of the model, the device will need testing on a broader selection of microorganisms involved in CLABSI, such as Klebsiella species, Pseudomonas aeruginosa, and Candida albicans, at different growth phases and after 48 h, to better determine the bacterial inhibition duration and effect, as well as assess HOCl production over time. e-catheters can produce HOCl if chloride ions are provided in the intraluminal solution (here, 0.9% NaCl); future experiments could assess when chloride ions are consumed and HOCl is no longer produced and therefore determine the ideal time to replace intraluminal solutions. In addition, further studies are warranted to assess the toxicity of HOCl on endothelial cells and blood cells and its reaction with different blood components and to assess its performance in an *in vitro* flow system. Ultimately, the described e-catheter prototype provides a proof of concept demonstrating antimicrobial activity of electrochemically generated HOCl to prevent intraluminal catheter infection. The prototype serves as a preliminary model to move from handmade devices to custom-built e-catheters in future testing.

In conclusion, the described e-catheter represents a novel *in vitro* system capable of electrochemical generation of intraluminal HOCl when polarized at 1.5 V_Ag/AgCl_, which shows preliminary antimicrobial activity against bacterial species commonly implicated in CLABSI.

## MATERIALS AND METHODS

### Catheter model.

**FIG 4:**
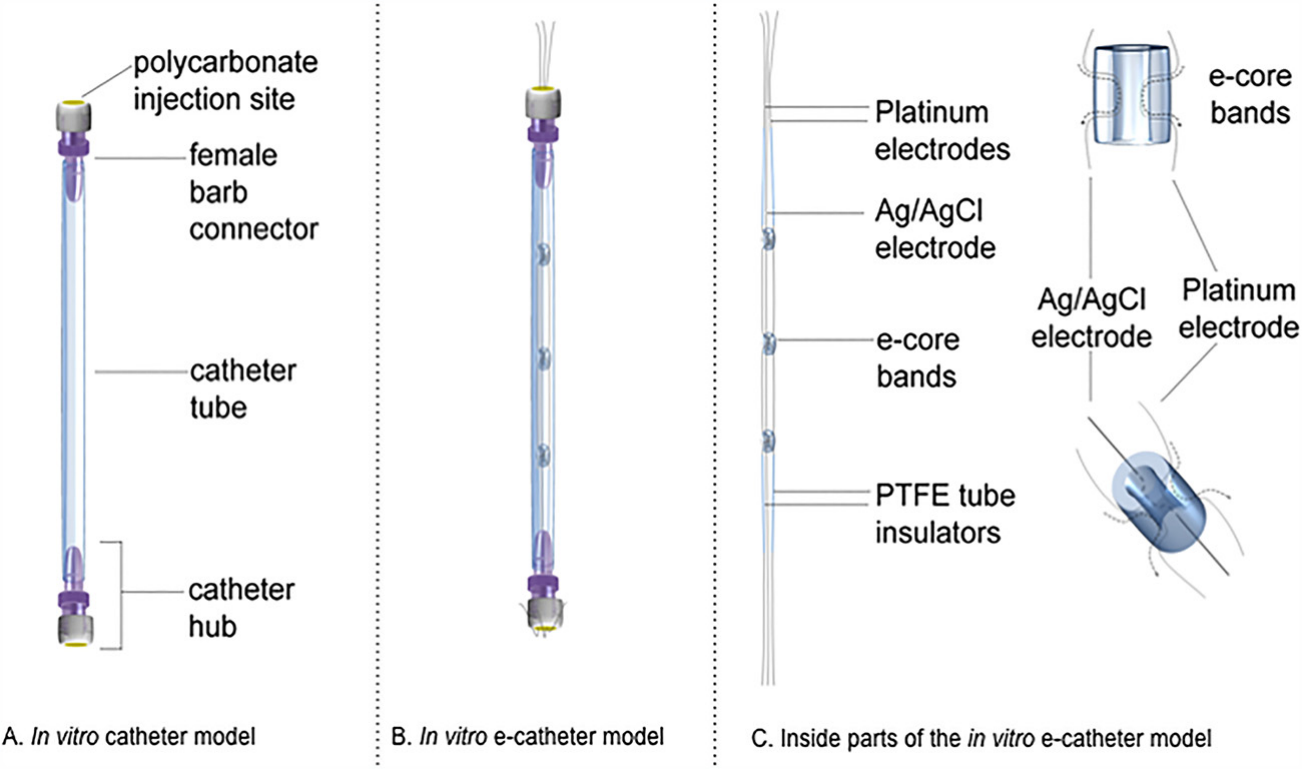
*In vitro* catheter and e-catheter models.

Catheters were built from 10-cm, 4-mm-inner-diameter Tygon S3 E-3603 catheter tubing (Fisher Scientific, Hanover Park, IL) with female Luer lock barb connectors (Qosina Corp., Ronkonkoma, NY) inserted on each end, capped with polycarbonate male Luer injection hubs (Qosina Corp., Ronkonkoma, NY) on each end (Fig. 4A).

### Electrochemical catheters.

E-catheters consisted of catheters (described above) in which two 25-cm (200-μm-diameter) platinum wires (referred to as working and counter electrodes) and one 25-cm silver/silver chloride-plated wire (Ag/AgCl, referred to as the reference electrode) were inserted. To prevent the three wires from touching one another during polarization, they were inserted through three 5-mm-long by 2.3-mm-diameter plastic tubes referred to as e-core bands (Fig. 4C). Wires at both extremes were insulated with a 32 AWG polytetrafluoroethylene (PTFE) extruded tube and passed through a polycarbonate male Luer injection hub, leaving exposed electrodes at both ends (Fig. 4B). The bottom injection hub was used to prevent leakage of intraluminal contents during experiments.

Catheters and e-catheters were inserted into uncapped 15-ml Falcon tubes (Fisher Scientific, Hanover Park, IL) to maintain them in an upright position during experiments. Catheters underwent autoclave sterilization at 121°C for 30 min with the upward hub open to avoid deformation due to buildup of pressure inside. After sterilization, catheters and e-catheters were filled with 1 ml sterile 0.9% NaCl using a 3-ml syringe inserted through the top injection hub, with a 25-gauge by 1.6-cm needle inserted simultaneously at the bottom injection site (to avoid intraluminal pressure buildup and remove air bubbles). After filling, the upward hub was closed and needles were removed. Preliminary electrochemical experiments were conducted using a Gamry G300 potentiostat (Gamry Instruments, Warminster, PA) to determine the potential range suitable for generating HOCl in the e-catheter in the absence of bacteria. Cyclic voltammograms were recorded while sweeping the working electrode potential from 0.0 V_Ag/AgCl_ to 2.0 V_Ag/AgCl_ and then back to 0.0 V_Ag/AgCl_ at a scan rate of 0.010 V/s. Three cycles were recorded for each experiment; the third cycle is reported as a representative data set. Cyclic voltammetry results were used to verify the potential at which anodic reactions occur. Chronoamperometric scans were recorded to measure the current magnitude, while the working electrode potential was controlled at 1.5 V_Ag/AgCl_.

### pH and HOCl measurement.

pH and HOCl measurements were performed in infected (see below) e-catheters after 48 h of polarization. The intraluminal solution was centrifuged for 10 min at 5,000 rpm (rpm) to remove planktonic bacteria. One hundred microliters of supernatant obtained after centrifugation of the intraluminal contents was applied to pH indicator strips (pH range, 4.0 to 7.0, ColorpHast; EMD Chemicals, Inc., Burlington, MA), and the pH defined using a colorimetric scale from the vendor.

A free chlorine test was used to determine HOCl concentrations in the intraluminal fluid (TNTplus 866; Hach, Loveland, CO). Chlorine (Cl_2_) is produced electrochemically via chloride oxidation, which dissociates in water to produce HOCl and hypochlorite (24). For each polarized e-catheter at 48 h (see below), 1 ml of the supernatant was added to 7 ml of sterile water to fill the test vials, and free chlorine was measured using a DR1900-01H portable spectrophotometer (Hach, Loveland, CO). Free-chlorine concentrations measured in milligrams per liter were first corrected by the dilution used to fill the test vials. Then, the HOCl concentration in micromolar was estimated according to the dissociation equilibrium of HOCl/OCl^−^ at the pH measured for each replicate (25). The lower limit of detection of the free-chlorine test for Cl_2_ was 0.05 mg/liter, corresponding to 5.42 μM HOCl at pH 6.14 (mean pH in experiments reported here).

### Evaluation of preventative antimicrobial activity of e-catheters.

To preliminarily test the antimicrobial prevention effect of the e-catheters, four clinical isolates derived from catheter-associated infections were studied, S. aureus IDRL-10296, S. epidermidis NRS34, E. faecium IDRL-11625, and E. coli IDRL-7343. Bacterial inocula were selected to target a concentration of >8 log_10_ CFU/ml after 48 h in blank catheters. An initial bacterial concentration of 10^4^ CFU/ml was used for S. aureus IDRL-10296, 10^7^ CFU/ml for S. epidermidis NRS34, 10^5^ CFU/ml for E. faecium IDRL-11625, and 10^4^ CFU/ml for E. coli IDRL-7343. Different inoculum sizes were used to establish 48-h concentrations that would allow determination of a 3-log reduction in bacterial concentrations between treatment groups.

Bacteria were subcultured from frozen aliquots onto BBL Trypticase soy agar (TSA II) with 5% sheep blood plates (Becton, Dickinson, Franklin Lakes, NJ) and incubated at 37°C overnight. One bacterial colony from this first plate was then subcultured on a second TSA II plate for 24 h. One to three colonies from this second plate were added to 2 ml of Trypticase soy broth (TSB) and incubated for 2 h at 37°C on an orbital shaker at 120 rpm to reach a 0.5 McFarland standard (∼1.5 × 10^8^ CFU/ml). The bacterial suspension was then diluted in TSB to the targeted bacterial inoculum concentration for each isolate (see above).

### Experimental setup.

Each experiment set comprised one catheter and two e-catheters filled with 1 ml 0.9% NaCl. The catheter referred as the “blank catheter” served as a control to assess any effect of platinum and Ag/AgCl wires, as described elsewhere (30). One e-catheter was unpolarized and served as a control for the polarized e-catheter. One e-catheter was polarized using a custom 4-channel potentiostat (31) and is referred to as “polarized”; the working electrode was polarized at a constant potential of 1.5 V_Ag/AgCl_ for 48 h at 37°C to generate HOCl. After 24 h, the blank catheter, nonpolarized e-catheter, and polarized e-catheter were inoculated with 100 μl of bacterial broth and incubated in an air incubator at 37°C for an additional 24 h. The final volume in each catheter was 1.1 ml.

### Intraluminal bacterial cell quantification.

After 48 h of polarization (i.e., 24 h after bacterial challenge), catheters were unplugged from the setup, hubs were uncapped in a sterile fashion, and catheters were forcefully flushed with 0.9 ml of sterile water and 2 ml of air into a sterile 15-ml Falcon tube using a 3-ml syringe. Intraluminal fluid was then centrifuged at 5,000 rpm for 10 min, and the supernatant was aspirated without disturbing the bacterial pellet at the bottom of the tube. The cell pellet was resuspended in 1 ml of sterile water and vortexed; quantitative cultures were performed using serial dilutions. Bacterial concentrations were calculated in log_10_ CFU/ml with an estimated lower limit of quantification of 1 log_10_ CFU/ml. For samples below this limit of quantification, 1 ml of TSB was added to the remainder of the intraluminal fluid, and growth was reported based on turbidity of the broth, bringing the limit of detection to 1 CFU/ml. Values were reported as 0 log_10_ CFU/ml if the broth culture was negative after 48 h.

### Statistical analysis.

Comparison of intraluminal bacterial cell concentrations at 48 h among the three experimental groups (polarized, nonpolarized, and blank catheters) was performed using the Kruskal-Wallis test for nonparametric samples. Bonferroni’s correction was not performed due to the small sample size. All experiments were performed in triplicate. Summary statistics are reported as the mean with standard deviation (for triplicates). All tests were two sided; *P* values of <0.05 were considered statistically significant. A clinically significant antimicrobial effect was defined as a >3 log_10_ CFU/ml mean reduction in bacterial concentrations compared to the blank catheter. This mathematically represents a 99.9% mean reduction in viable bacterial cell count (32). Analyses were performed using GraphPad Prism version 8.0.

### Data availability.

Data from this study are available from the corresponding author.
