# Supplementary material for: Hypochlorous Acid-Generating Electrochemical Catheter Prototype for Prevention of Intraluminal Infection
Source: Microbiol Spectr. 2021 Oct 27;9(2):e00557-21. doi: 10.1128/Spectrum.00557-21 (PMC8549727; doi:10.1128/Spectrum.00557-21)
Supplement: SUPPLEMENTAL FILE 1 — Supplemental material. Download SPECTRUM00557-21_Supp_1_seq7.pdf, PDF file, 0.1 MB [file spectrum00557-21_supp_1_seq7.pdf]

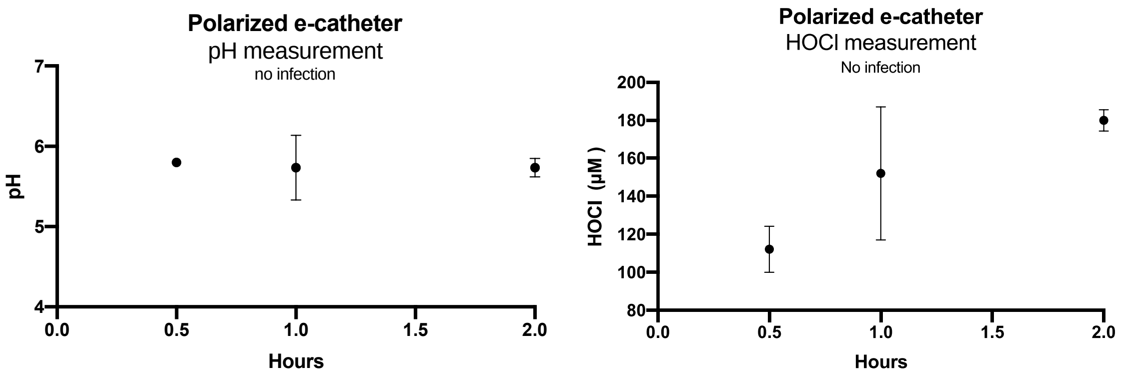


**Supplemental Figure S1.** HOCl and pH measurements without infection at 0.5, 1 and 2 hours.

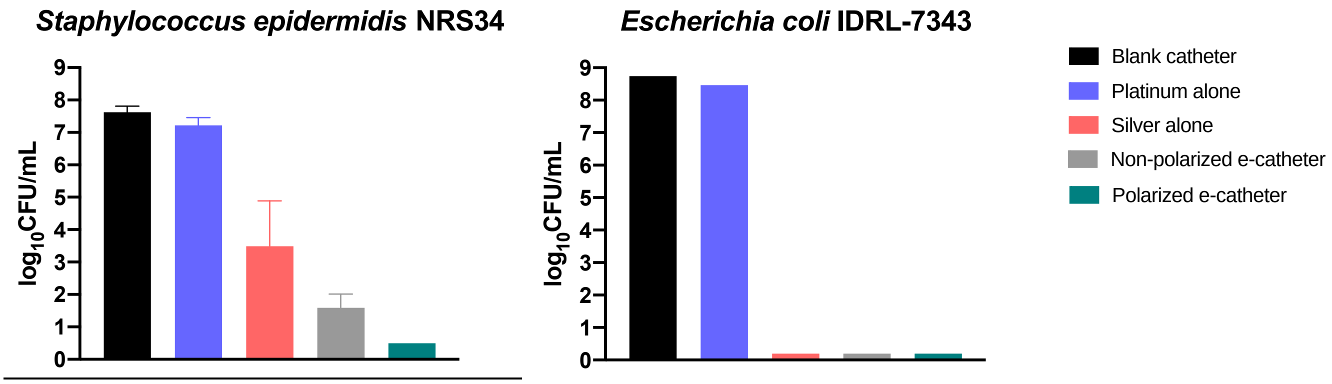


Error bars represent standard deviation if experiments were done in at least duplicates.

**Supplemental Figure S2.** Effect of polarized and non-polarized e-catheters and different components on bacterial cells with 48 hours of exposure.
